# Comparing DNA Methylation Landscapes in Peripheral Blood from Myalgic Encephalomyelitis/Chronic Fatigue Syndrome and Long COVID Patients

**DOI:** 10.3390/ijms26146631

**Published:** 2025-07-10

**Authors:** Katie Peppercorn, Sayan Sharma, Christina D. Edgar, Peter A. Stockwell, Euan J. Rodger, Aniruddha Chatterjee, Warren P. Tate

**Affiliations:** 1 Department of Biochemistry, School of Biomedical Sciences, University of Otago, Dunedin 9016, New Zealand; katie.peppercorn@otago.ac.nz (K.P.); tinaedgar24@gmail.com (C.D.E.); 2 Department of Pathology, Dunedin School of Medicine, University of Otago, Dunedin 9016, New Zealand; shasa470@student.otago.ac.nz (S.S.); peter.stockwell@otago.ac.nz (P.A.S.); euan.rodger@otago.ac.nz (E.J.R.)

**Keywords:** Myalgic Encephalomyelitis/Chronic Fatigue Syndrome, long COVID, DNA methylation, peripheral blood mononuclear cells, differentially methylated fragments, hypomethylation, hypermethylation

## Abstract

Post-viral conditions, Myalgic Encephalomyelitis/Chronic Fatigue Syndrome (ME/CFS) and Long COVID (LC), share > 95% of their symptoms, but the connection between disturbances in their underlying molecular biology is unclear. This study investigates DNA methylation patterns in peripheral blood mononuclear cells (PBMC) from patients with ME/CFS, LC, and healthy controls (HC). Reduced Representation Bisulphite Sequencing (RRBS) was applied to the DNA of age- and sex-matched cohorts: ME/CFS (*n* = 5), LC (*n* = 5), and HC (*n* = 5). The global DNA methylomes of the three cohorts were similar and spread equally across all chromosomes, except the sex chromosomes, but there were distinct minor changes in the exons of the disease cohorts towards more hypermethylation. A principal component analysis (PCA) analysing significant methylation changes (*p* < 0.05) separated the ME/CFS, LC, and HC cohorts into three distinct clusters. Analysis with a limit of >10% methylation difference and at *p* < 0.05 identified 214 Differentially Methylated Fragments (DMF) in ME/CFS, and 429 in LC compared to HC. Of these, 118 DMFs were common to both cohorts. Those in promoters and exons were mainly hypermethylated, with a minority hypomethylated. There were rarer examples with either no change in methylation in ME/CFS but a change in LC, or a methylation change in ME/CFS but in the opposite direction in LC. The differential methylation in a number of fragments was significantly greater in the LC cohort than in the ME/CFS cohort. Our data reveal a generally shared epigenetic makeup between ME/CFS and LC but with specific, distinct changes. Differences between the two cohorts likely reflect the stage of the disease from onset (LC 1 year vs. ME/CFS 12 years), but specific changes imposed by the SARS-CoV-2 virus in the case of the LC patients cannot be discounted. These findings provide a foundation for further studies with larger cohorts at the same disease stage and for functional analyses to establish clinical relevance.

## 1. Introduction

The COVID-19 pandemic has infected a reported ~778 million people worldwide [1], and from those have arisen an estimated 60 million cases or more of a post-viral debilitating syndrome generally referred to as Long COVID (LC) [2,3,4] and clinically as Post-Acute Sequelae of SARS-CoV-2 infections (PASC) [5]. This scenario is unique as cases of post-viral syndromes in the past have been ‘drip fed’ from geographically isolated boutique infectious outbreaks, or endemic viruses in our communities, like Epstein-Barr virus, or influenza. Such post-viral conditions over the last 100 years have been given a plethora of different names, but from about 2016, the common name of Myalgic Encephalomyelitis/Chronic Fatigue Syndrome (ME/CFS) has settled to encompass them all. Derived from two isolated infectious disease outbreaks, first in London (ME) [6] and second in Incline Village, Nevada (CFS) [7], it has been accepted as documenting the same illness.

LC has arisen from the huge case numbers of COVID-19 from the single triggering virus, SARS-CoV-2, and that has meant a sudden huge case burden of LC, as distinct from the slow build-up of cases of ME/CFS from the multiple triggers, boutique outbreaks, endemic viruses, or other major stressors. Since experience has shown that the large majority of patients diagnosed with ME/CFS have the condition as a lifelong illness, the absolute numbers of worldwide cases slowly increase with each new case [8]. The LC onset scenario awakened the clinical and scientific communities to the severely debilitating nature of these diseases. It has highlighted the lack of prior investment in research into understanding the ME/CFS post-viral/stressor syndrome. ME/CFS patients have been coined the ‘missing millions’ in protest that the impact of their condition on their lives has been met with inadequate recognition and support from clinical and social services [9]. The hope is that the current focus and large research investment in LC will benefit both ME/CFS and LC patients in finding management solutions and treatments to improve their quality of life. A reversal of the debilitating symptoms with a new understanding of these conditions might be possible, given the evidence that the neurological dysfunctions are not neurodegenerative.

For the 5–10% of the population who have genetic or co-morbidity susceptibility to develop LC and ME/CFS [2,10], new COVID infections will continue to generate these debilitating ongoing post-viral conditions at a significant frequency. Recent data from Fang et al. [11] showed rates of LC were similar in unvaccinated (8.4%) and people vaccinated with two doses (8.7%) but reduced in those who had had a third vaccination booster (5.8%). A study that examined the records of >400,000 US veterans who had the infection during the period March 2020 to February 2022 at one year post infection in the unvaccinated group the rates declined from 9.5% (delta variant) to 7.7% (omicron variant), and in the vaccinated the rates were 5.3% (delta) and 3.5% (omicron) [12]. Nevertheless, even with these lowered rates, newly COVID-infected people are still developing LC, with the virus now becoming endemic, adding constantly to the global health burden.

Strong linkage of genetic factors to the susceptible group who develop the post-viral/stressor conditions has recently been discovered, after Precision Life (Oxford) added a combinatorial platform to GWAS technology that allowed combinations of single-nucleotide polymorphisms (SNPs) to be evaluated. It showed small clusters of SNPs in combination are linked to ME/CFS [13] and LC [14]. For example, in the ME/CFS study, 199 SNPs in 15 clusters of 84 communities each with 3–5 specific variations were linked to 14 genes, relevant to cellular mechanisms dysfunctional in ME/CFS. These genetic variations could account for 91% of the samples from ME/CFS patients in the United Kingdom Biobank. Long COVID with a more diverse cohort gave more complex results—with 73 genes linked to the condition, but 39 of the SNPs detected were also linked to nine of the genes identified in the ME/CFS study [14].

A key question is whether what is known about ME/CFS provides a suitable model for insight into understanding the ME/CFS-like syndrome subgroup of LC that represents ~50% of the cases [15,16]. Conversely, will new research findings emerging from studies of LC, like the NIH’s RECOVER initiative [17], benefit ME/CFS patients? Underpinning these questions is whether the physiological responses in susceptible people to the SARS-CoV-2 virus are similar or significantly different from those responses in ME/CFS patients who have had a diverse range of stress triggers leading to their ME/CFS. The current study aims to provide evidence on this question.

There is a wide array of >200 symptoms reported by both groups of LC and ME/CFS patients, of which >95% overlap, suggesting high similarity at least in the clinical phenotypic effects on the patients [18,19]. Indeed, several authors have referred to LC as being appropriately renamed ME/CFS if it lasts beyond two years, for example [3]. LC has been with us for only five years, and it might present biologically in research studies currently as a juvenile form of ME/CFS, since the mean time of those affected with ME/CFS and being studied is generally from onset up to ten years and beyond. However, a wide range of biological dysfunctions is already known to be common to the two conditions [10]. In 2022 Komaroff listed many: dysautonomia, generation of autoantibodies, switch in energy generation, low energy/hypometabolic state, increased oxidative stress, mast cell activation, abnormal cardiopulmonary responses to exercise tests, blood vessel/coagulation disturbances, Epstein Barr virus and other herpes viral reactions, small fibre neuropathy, cognitive dysfunction, disturbed HPA axis, neuroinflammation, and gut microbiome changes [20]. Importantly, those LC patients affected with the ME/CFS-like syndrome experience the same core defining symptom of ME/CFS, post-exertional malaise, but with some differences in its expression that were attributed to the early stage of the condition in LC [21].

Our recent comparative study of immune cell protein dysregulation in LC and ME/CFS identified a group of differentially regulated proteins common to both ME/CFS and LC patients. In most cases, the proteins were up- or down-regulated similarly, but in a minority of cases, the differential regulation occurred in opposite directions. Similar molecular pathways were affected, for example, mitochondrial functions linked to energy production, immune system processes, and cytokine regulation. Major overlapping clusters in both cohorts were found among proteins related to gene expression. An overarching conclusion from this study was that LC and ME/CFS have similar major dysfunctions in their immune cell physiology and energy production [22].

A recent comparative investigation of both adaptive and innate immune dysregulation underlying ME/CFS and long COVID highlighted the potential importance of immune exhaustion in disease progression. Differential gene expression analysis in ME/CFS showed downregulated IFN signalling and immunoglobulin gene expression, indicative of immune suppression, and pathway analysis implicated dysregulated macrophage activation, cytokine production, and immunodeficiency signalling. Long COVID patients had antigen presentation, cytokine signalling, and immune activation dysregulated, with differentially expressed genes also associated with B cell development, macrophage activation, and cytokine signalling [23].

Comparative genome-wide DNA methylation studies in ME/CFS and LC have not yet been carried out, but there are studies on ME/CFS patients utilizing mononuclear immune cells or specific immune cell subtypes and using either Reduced Representation Bisulphite Sequencing (RRBS) [24,25] or Illumina Methylation 450K/EPIC arrays [26,27,28,29,30]. Our first RRBS-based analysis of ME/CFS patients identified 76 differentially methylated fragments and 394 differentially methylated cytosines linked to 122 genes involved in neurological, immune, and metabolic pathways [24]. A ME/CFS relapse-recovery longitudinal study we conducted on two ME/CFS patients and a matched control identified 577 differentially methylated fragments during the relapse with significant involvement in IL-8 signalling, NFκB dysregulation, and neutrophil degranulation. The methylation changes at individual sites reverted to the levels prior to the relapse when the relapse resolved [25].

In this current comparative study of LC and ME/CFS, we have examined the DNA methylome landscape of age/sex linked LC and ME/CFS patient cohorts and healthy controls.

## 2. Results

### 2.1. Whole-Genome DNA Methylation Patterns

**Figure 1:**
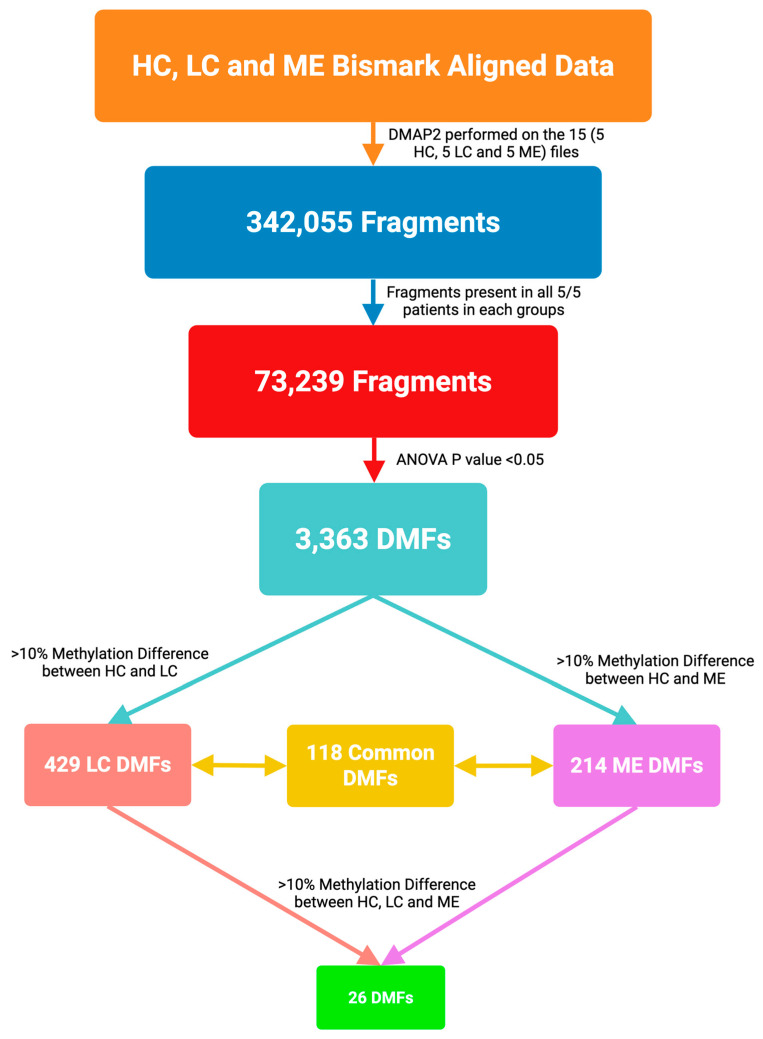
Flow diagram of the analysis of the DMFs in the ME/CFS, LC cohorts compared with age/sex matched HCs. Bam files were created from the Reduced Representation Bisulphite Sequencing (RRBS) data, the 15 samples analysed by DMAP2 [31] to give 342,055 methylated fragments, those fragments found in all five patients of each of the three cohorts made were sub-selected (73,239 fragments), and those significantly different (*p* < 0.05) comparing the ME/CFS and LC cohorts with HCs identified (3363 DMFs). Applying a limit of 10% change in differential methylation from the HCs gave 429 LC DMFs and 214 ME/CFS DMFs, of which 118 were in common between the two disease cohorts. Twenty-six of these 118 had a >10% methylation difference between the two patient cohorts.

Genome-scale DNA methylation changes were derived from the samples from each of five Healthy Controls (HC), five ME/CFS patients, and five LC patients. There were 342,055 fragments that covered 2,151,222 CpG sites in the 15 samples, from a total of 3.1 × 10^8^ sequence reads (9.2 × 10^7^ for HC, 10.8 × 10^7^ for ME/CFS, and 10.9 × 10^7^ for LC), allowing for comprehensive genome-wide analysis (Table S1). The global methylation genomic patterns were assessed, and the median value obtained for global methylation with all three cohorts was similar (median methylation = 89.3% for HC, 89.9% for ME/CFS, and 88.7% for LC), showing hypermethylation was predominant. The promoter regions were, by contrast, predominantly hypomethylated in all three cohorts (Table S2). The intronic and exonic regions were predominantly hypermethylated with a similar median in all cohorts and with a slight increase in the first quartile in the ME/CFS cohort. A flow diagram of the decisions for analysis of the DMFs between ME/CFS and LC is shown in Figure 1.

**Figure 2:**
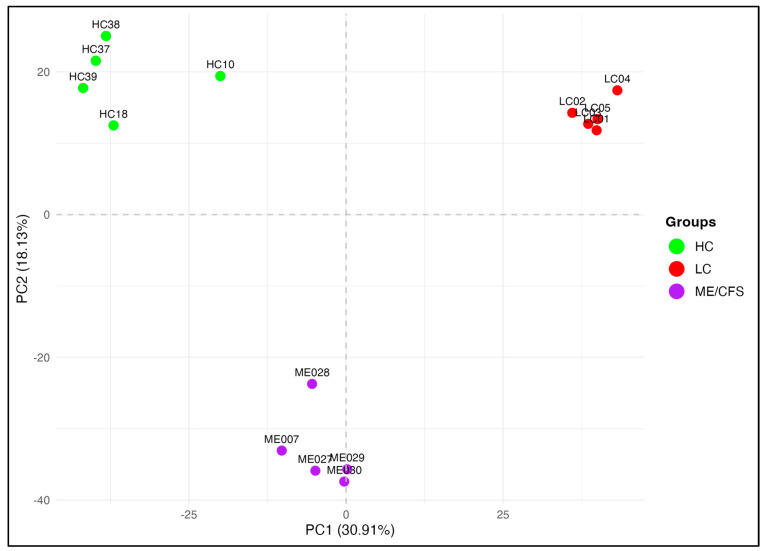
PCA of differentially methylated fragments in all members of the HC, LC, and ME/CFS cohorts. PCA plot illustrating three distinct clusters representing HC, ME/CFS, and LC based on 3363 DMFs common to all cohorts filtered by *p* < 0.05, without considering methylation difference.

To begin the evaluation of the methylation landscape, the fragments were first sub-selected with a strict criterion as being present in all five patients of each of the three cohorts, and 73,239 fragments were then available for further analysis (Figure 1). A Principal Component Analysis (PCA) separated the cohorts into three distinct clusters (Figure 2). This indicated that both the ME/CFS and LC cohorts showed differential methylation within these common fragments compared with the healthy controls. The tight clustering of all patients of each cohort in the plot suggested most of the DMFs and their characteristics were common to all patients within the cohort. The separation of the ME/CFS and LC cohorts into individual clusters (Figure 2) suggested that there might be differences between the extent of the methylation change at specific sites between the two cohorts, or changes at specific sites in only one of the disease cohorts. For this analysis, a significance of *p* < 0.05 was imposed for the methylation change, but no limits on the degree of methylation change and 3363 fragments met that criterion.

### 2.2. Characteristics of the Differential Methylation Changes in LC and ME/CFS

**Figure 3:**
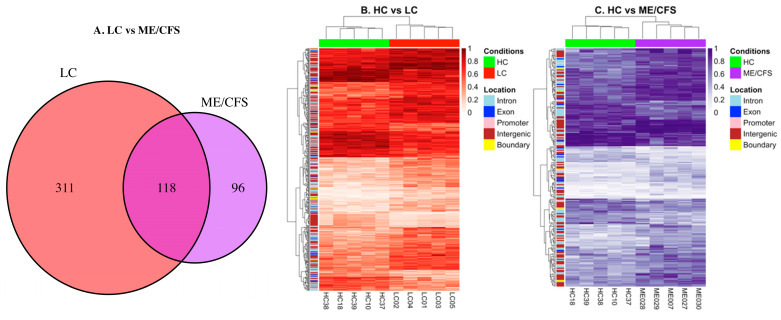

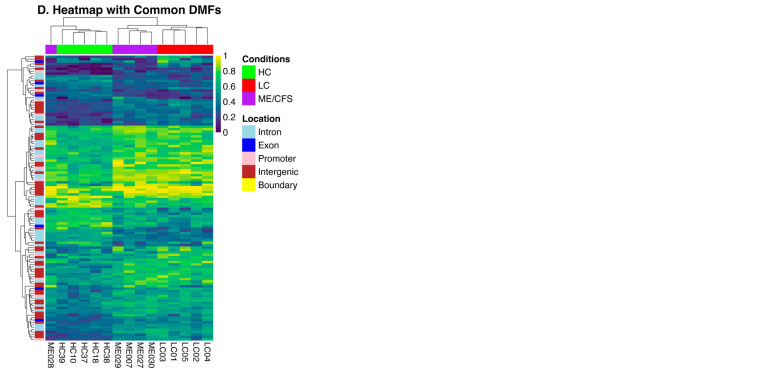
Heatmaps of differential methylation on fragments in the LC and ME/CFS patient cohorts compared with HCs. (**A**) Venn diagram showing overlapping DMFs in LC and ME (*p* < 0.05, >10% methylation difference). Heatmaps show DMFs between (**B**) LC and HC, (**C**) ME/CFS and HC (*p* < 0.05, >10% methylation difference). The annotation column bar at the top of each heat map represents the cohort group (LC-red, HC-green, ME/CFS-purple), and the annotation on the Y axis shows the genome region of the DMF (colour coded in the key). The colour gradient shown in the key indicates the methylation level of the fragments, with the darker colours representing the higher degree of methylation. The locations of the DMFs are annotated beside the heatmaps, gene promoters (−1 kb to +5 kb from the TSS), exons, introns, and intergenic elements (>5 kb upstream from the nearest TSS), and intron-exon boundary elements. (**D**) Heatmap showing the methylation values of the 118 common DMFs in the ME/CFS and LC cohorts and illustrating the methylation differences in the LC and ME/CFS patient groups compared with the HC group. The annotation column bar at the top of the heat map represents the cohort groups (LC-red, HC-green, ME/CFS-purple), and the annotation row bar on the left side shows the genome region of the DMFs (colour coded in the key). The colour gradient from yellow to indigo shown in the key ranges from hypermethylation (yellow) to hypomethylation (indigo). It indicates the colours matching specific methylation values.

The analysis identified 429 DMFs between LC vs. HC (Table S3, Figure 1) and 214 DMFs between ME/CFS vs. HC (Table S4, Figure 1) (*p*-value < 0.05, minimum 10% methylation difference). Of the 429 DMFs between LC and HC, 148 were hypomethylated and 281 were hypermethylated, and among the 214 DMFs between ME/CFS and HC, 69 were hypomethylated and 145 were hypermethylated. Thus, both cohorts showed methylation patterns shifting towards hypermethylation when compared to HCs, and in this study, LC patients show more abundant methylation changes than the age/sex matched ME/CFS patients. Of the 429 DMFs in LC and 214 DMFs in ME/CFS when compared to HC, 118 DMFs were common to both disease datasets, as shown in the Venn diagram (Figure 3A). The Pearson R score was 0.88 when the methylation data within the common fragments of the two cohorts were compared, indicating a high correlation between the data at the differentially methylated sites from the LC and ME/CFS cohorts and emphasizing the similarity between the two conditions. Heat maps of LC vs. HC and ME/CFS vs. HC are shown in Figure 3B,C. Unsupervised hierarchical clustering of DMFs between HCs and LCs (Figure 3B) and between HCs and ME/CFS (Figure 3C) has separated the differential methylation patterns so that all members of each of the three cohorts (HC-green, LC-red, or ME/CFS-purple) group together. The regions of the genome from which the DMFs are derived (intergenic, promoter, exon, intron, or the boundary between intron and exon) are shown on the axis at left, colour coded in the key. The heatmaps showed distinct patterns of the DMFs: (i) most hypermethylated fragments in the HC cohort became more strongly hypermethylated in both LC and ME/CFS cohorts, and a minority became more hypomethylated; (ii) most hypomethylated fragments in the HC generally also became more hypermethylated but with a minority becoming more hypomethylated. This was a common pattern for both disease cohorts. The minority group of DMFs was larger in the LC cohort than in the ME/CFS cohort (iii). Some fragments with mid-range methylation were more variable in their methylation among the individual members of the HC cohort but became predominantly more hypermethylated in the patient cohorts, again with a minority showing greater hypomethylation. A heatmap illustrating the methylation values and genomic location of the 118 common fragments (Table S6) found in each cohort compared with the HC cohort was generated and showed that the majority of these DMFs are similarly differentially methylated (Figure 3D). In contrast to the heat maps in Figure 3B,C, unsupervised hierarchical clustering of DMFs has separated one ME/CFS patient (ME028) in the ME/CFS group from the other ME/CFS members into a ‘group of one’ whereas all five members of each of the other two cohorts (HC-green, LC-red) group together. It is interesting to note that in the PCA analysis shown in Figure 2, this patient, ME028, was more widely separated from the other members of the ME/CFS cluster. The heat map showed a wide range of methylation levels in the HC of the 118 fragments and both changes towards hypermethylation and hypomethylation in the LC and ME/CFS cohorts.

### 2.3. DMFs Associated with Gene Promoters and Gene Exons

**Figure 4:**
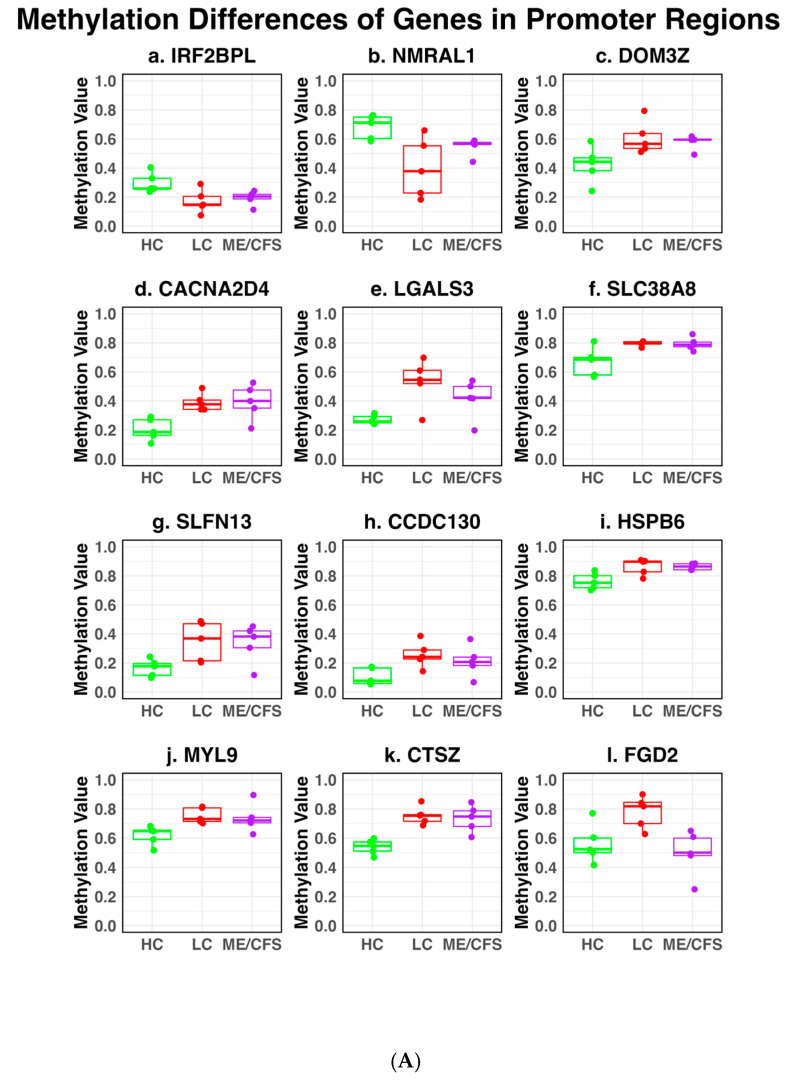

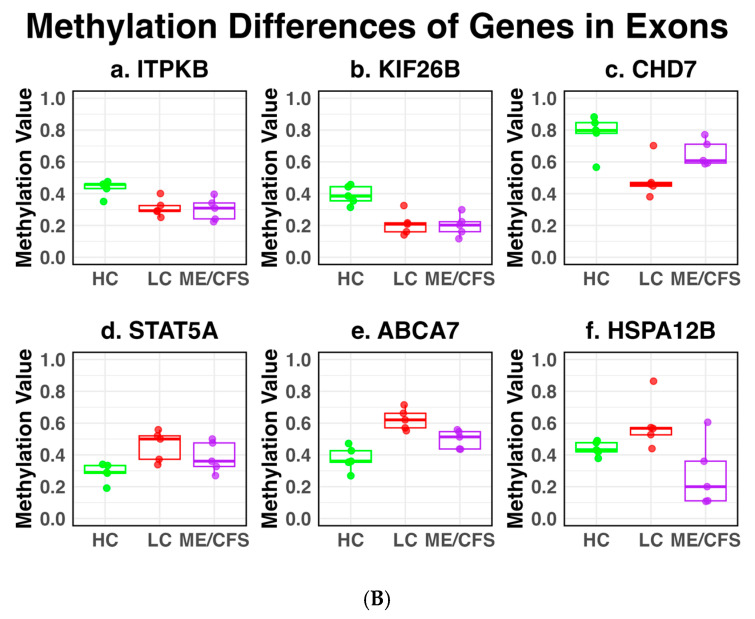
Box plots showing individual patient differential methylation characteristics at gene-associated sites. Box plots of (**A**) DMFs associated with gene promoters; (**B**) DMFs associated with gene exons within the 118 fragments in common between LC and ME/CFS patients. The plots show the individual patient values of the LC, ME/CFS, and the HC cohorts. In both (**A**,**B**), the HC is coloured green, the LC red, and ME/CFS purple. Individual patients are indicated by the points on the plots. The medians are indicated by the bold line, and the box indicates the first and third quartiles. Associated gene IDs are displayed at the top of each sub plot.

Among the 118 common DMFs, twelve were associated with *gene promoters*. Nine (associated genes-*LGALS3, SLC38A8, SLFN13, CCDC130, HSPB6, CTSZ, MYL9, DOM3Z, CACNA2D4*) showed hypermethylation in both LC and ME/CFS, and two (associated genes -*IRF2BPL, NMRAL1*) were hypomethylated in both conditions. In contrast, one, associated with *FGD2,* was hypermethylated in the LC patients and relatively unchanged in the ME/CFS patients when compared with healthy controls. Six fragments were associated with *gene exons* (*ITPKB, KIF26B, CHD7, STAT5A, ABCA7, HSPA12B*) among the 118 common DMFs. Three (*ITPKB, KIF26B, CHD7*) were hypomethylated in both the LC and ME patients, and two (*STAT5A, ABCA7*) were hypermethylated in both conditions. By contrast, one *HSPA12B* showed hypermethylation in LC and hypomethylation in ME/CFS compared to HC (Table S5). The twelve identified DMFs in promoter regions associated with specific genes are shown as box plots in Figure 4A, indicating the individual values of the five patients from each disease cohort and the controls. In most cases, the changes were found in all patients of the cohort as inferred from the PCA plot (Figure 2), and variation among the individual patients was relatively small, apart from two sites for the LC cohort. The HC median values of the two promoter sites that were hypomethylated in both cohorts (*IRF2BPL and NMRAL1*) were significantly different, 25% and 75% methylation, respectively. For the eight sites that were hypermethylated in both LC and ME/CFS compared with HCs, the median values of the methylation in the HCs varied from 9–75%. The promoter site associated with *FGD2* that was hypermethylated only in the LC patients compared with the HC increased from 52% to 81% whereas it was unchanged at 50% in the ME/CFS cohort.

The box plots of the six DMFs associated with exons of specific genes, *ITPKB, KIF26B, CHD7, STAT5A, ABCA7, and HSPA12B*, are shown in Figure 4B. Most had HC methylation median values of ~40%. The variations in the methylation values among individual patients, while greater than in the controls, were still relatively small.

### 2.4. Methylation Differences Between Long COVID and ME/CFS

There were also differences in the *degree* of methylation change between the two disease cohorts. There were 26 DMFs among the 118 DMFs with >10% methylation difference between LC and ME/CFS (Table S6). Fifteen of the 26 DMFs lie within the intergenic regions, and 11 are in more defined gene regions and therefore easier to interpret. Of these 11 DMFs, three of the genes correspond to sites within exons (*CHF7, ABCA7, and HSPA12B*) and three within promoter regions (associated with *FGD2, NMRAL1, and LGALS3*).

In Table 1, the chromosome and the genomic start and end sites of the 26 DMFs where there was a >10% difference in the methylation changes between the LC and ME/CFS cohorts are shown. The genomic locations of the DMFs (promoter, exon, intron, or intergenic) are documented, and the percentage differences in methylation between LC vs. HC, ME/CFS vs. HC, and then LC vs. ME/CFS are indicated. GeneIDs linked to differentially methylated promoters, exons, and introns are given, but not the possible gene linkages for intergenic regions. The DMF changes between the two cohorts are sorted according to the degree of difference between the LC and ME/CFS (−29% to +37%-shown in last column). It is to be noted that the exons and promoters in Table 1 are already represented in the box plots in Figure 4A,B.

In 8 of the 26 DMFs where there was hypomethylation in both cohorts, it was greater in seven in the LC patient group, and in only one fragment in the ME/CFS patient group. Of the 12 of the 26 DMFs that were hypermethylated in both cohorts, nine showed greater differential methylation in the LC cohort and three in the ME/CFS cohort. Hence, the LC group of patients generally has a greater change in their methylation status in 16 of the 20 DMFs compared with the ME./CFS patients when the change is in the same direction. By contrast, six of the 26 DMFs showed opposite changes in their differential methylation in the two cohorts (five hypermethylated in LC but hypomethylated in ME/CFS; one hypermethylated in ME/CFS and hypomethylated in LC).

### 2.5. Functional Pathway Analysis of the DMFs of Long COVID and ME/CFS

Among the 429 DMFs from HC vs. LC and 214 DMFs between HC vs. ME, the unique associated GeneIDs were isolated and tabulated, except for sites within the intergenic regions where gene linkages are much less certain. There were 215 unique genes linked to LC (Table S8) and 111 genes linked to ME/CFS (Table S9). To identify the functional pathways associated with the DMFs in LC and ME/CFS, pathway enrichment analysis was performed using Metascape [32]. This analysis revealed several shared pathways between the two conditions (Figure S1A,B), suggesting common biological mechanisms. Notably, Response to Wounding (GO:0009611) emerged as a key pathway involved in tissue repair and inflammatory processes, aligning with the immune dysregulation and chronic inflammatory responses observed in both conditions. Additionally, Regulation of System Process (GO:0044057) was significantly enriched, highlighting disruptions in physiological processes such as circulation and metabolism that may contribute to cardiovascular and neurological dysfunctions. Cellular Response to Cytokine Stimulus/Acid Chemical (GO:0071345/GO:0071229) was identified, which likely reflects the important role in immune signalling and inflammation, both of which are known to be altered in ME/CFS and LC. Furthermore, Regulation of Small GTPase-Mediated Signal Transduction (GO:0051057/GO:0051056) was enriched, implicating intracellular signalling pathways that modulate immune responses, cell migration, and tissue repair. Growth Regulation (GO:0040007/GO:0040008) was a prominent pathway, suggesting that aberrant growth signalling could contribute to impaired tissue regeneration in both conditions. Other than these similarities, the functional pathway analysis showed blood vessel morphogenesis, muscle organ development, AGE RAGE pathway, Neutrophil degranulation as other notable pathways in LC, whereas thyroid hormone production, leukocyte differentiation, negative regulation of T cell receptor pathway, heart development, and blood circulation were highlighted in ME/CFS.

## 3. Discussion

Although a preliminary patient study, the findings described here provide deep insights into the genome-wide DNA methylation landscape of LC and ME/CFS, consistent among the patients in each cohort, and highlight both important similarities as well as differences between these conditions. The latter may reflect simply the differences in the time from onset of each condition in the patients, with the LC group at one year still in the acute stage as a ‘juvenile’ form of the more well-established ME/CFS, here represented by chronic stage patients. A proportion of the differences in LC may reflect the specific characteristics of the viral trigger associated with the SARS-CoV-2 virus. This virus likely invokes unique molecular responses as well as similar responses to the other ME/CFS-causing stressors within the broad categories of dysfunctional physiology but still supports a very similar clinical phenotype. This can be explored in future studies matched not only by age and sex but also by time of onset.

Principal Component Analysis (PCA) of differentially methylated fragments identified in all patients of the three cohorts showed distinct clustering of the HC, LC, and ME/CFS cohorts, demonstrating that despite LC and ME/CFS having many changes in methylation at genomic sites in common, the global DNA methylation patterns can separate the two disease groups from each other as well, and both are well separated from the HC group. However, the extent of differential methylation was generally more pronounced in LC than in ME/CFS, with LC patients showing a greater number of differentially methylated fragments (DMFs) when compared to HCs. This can explain why the ME/CFS and LC cohorts cluster separately. The maximum length of time possible for LC cohorts since the start of the pandemic have had their condition is now 5–6 years compared with ME/CFS cohorts of 40–50 years or even more, and so future studies with these ME/CFS-like conditions through the disease stages will help to understand these apparent differences better. For example, the epigenetic DNA methylations changes may be more dynamic at earlier stages, whereas at later stages, as with the age/sex matched ME/CFS cohort in this study, there may be a lowered expression of the initial changes leading to the chronic debilitation in patients that has become more stable with time.

The hierarchical clustering of the methylation changes in the 118 DMFs shared between LC and ME/CFS demonstrated a high degree of correlation (Pearson R = 0.88), indicating substantial overlap between the two conditions. Interestingly, in the PCA reflecting the global methylation patterns of 3363 DMFs, *p* < 0.05 was present in all patients of the three cohorts (Figure 2), one ME/CFS patient, ME028, while still within the cluster, was more separated from the other patients. This patient was also separated from the other ME/CFS patients in the unsupervised clustering shown in the heat map of Figure 3D. It is the youngest patient in the cohort, and the onset to study time was much lower than the others in the ME/CFS cohort. It is well documented that the DNA methylome can change with age among all cultural groups [33]. In this study, we had one example of an older age/sex matched ME/CFS and LC patients and HC compared with the other 4 patients of each cohort (see Table 3-Methods). Interestingly, the older healthy control, HC10, in the PCA of Figure 2 is separated from the other HCs. However, in contrast, the two matched older ME/CFS and LC patients are tightly clustered within the other patients of their cohorts, suggesting the changes in the DNA methylome caused by the disease are more influential than those reflecting age.

The shared 118 DMFs in the ME/CFS and LC patients were primarily linked broadly to immune regulation and metabolic pathways, further supporting the hypothesis that LC and ME/CFS share common biological mechanisms. Table 2 shows the characteristics of the 15 DMFs that could be confidently linked to specific genes (i.e., in eight promoters and seven gene bodies) that were shown in Box plots with individual patients’ values in Figure 4A,B. Those in intergenic regions were not included here as connections to specific genes could only be tentatively inferred.

At nine of the twelve promoters, the degree of change was similar for the ME/CFS and LC patients, whereas in the other three promoters, there was a much greater change in the methylation of the LC patients. Nine promoters exhibited hypermethylation, whereas two were further hypomethylated. In one promoter, the changes were in opposite directions, hypermethylation for the LC patients and hypomethylation for the ME/CFS patients. For the six common DMFs in gene exons, five had similar degrees of change in both ME/CFS and LC patients, with three showing much greater changes in the LC patients, and one of those sites was more hypermethylated in LC but conversely more hypomethylated in ME/CFS.

Among the genes that were linked to the specific twelve gene promoters (Table 2), the gene product of the intron-less gene *IRF2BPL* is deduced to be an interferon regulatory factor 2 binding protein involved in central nervous system function [34] NMRAL1 is a redox-sensitive protein that also negatively regulates the activity of NF-kB, implicated in regulating the immune response and inflammation [35]. LGALS3 is also involved in inflammation and innate immunity, important for macrophage adhesion and T cell regulation [36]. *SLC38A8* is a gene responsible for a Na^+^ coupled neutral amino acid transporter with a preference for the neurotransmitter glutamate [37]. *SLFN13* is a gene whose gene product is an RNASE engaged in translational control and can inhibit a wide range of viruses [38]. CCDC130 protein is a likely mRNA splicing factor with an integral part in the function of the spliceosome [39]. HSPB6 is a heat shock protein, with a role in platelet function, which is likely to play a role in human muscle function [40], and CTSZ is a cathepsin in the family of cysteine proteases, reported to be protective in inflammatory gastric disease [41]. *MYL9* encodes a myosin regulatory light chain that may regulate muscle contraction [42], and CACNA2D4 is a voltage-gated calcium channel alpha(2)delta-4 subunit [43]. FGD2 is expressed in B lymphocytes, macrophages, and dendritic cells and affects antigen uptake, antigen presentation, and cell migration [44]. DOM3Z gene product has a role in pre-mRNA quality control and has been connected with the autoimmune disease, Systemic Lupus Erythematosus [45] Thus, the differential methylation of the twelve gene promoters affects genes that have functions that are related directly or indirectly related to immune functions, a key feature of the pathophysiology of the LC and ME/CFS conditions.

In case of the six differentially methylated exons (Table 2), the specific genes have more specific diverse functions. The gene product ITPKB is involved in signal transduction with calcium release in the endoplasmic reticulum related to cytokine production in T cells [46], and the KIF26B protein is an intracellular motor protein transporting organelles along microtubules [47]. *CHD7* encodes a helicase DNA-binding protein involved in transcriptional regulation [48], while STAT5A is a gene product that is critical for signal transduction and activation of transcription, mediating cellular responses to cytokines for proliferation, differentiation, and apoptosis [49]. ABCA7 protein is an ATP-binding cassette (ABC) lipid transporter linked to an increased risk of Alzheimer’s disease [50,51]. The *HSPA12B* gene is an atypical heat shock gene thought to be essential for angiogenesis and cell migration [52]. These findings suggest that changes to the expression of specific regulatory factors, identified as related to the immune system in some cases, may act as drivers of the distinct disease phenotypes of both LC and ME/CFS.

Genes associated with the ME/CFS DMFs of this study (HC vs. ME) were compared with those of our previous study of Helliwell et al., 2020 [23], where DMFs were identified between HC and ME (>15% mean methylation difference). The comparison identified genes in common, mostly linked from introns, but the methylation changes were in fragments at different sites within the introns in the two studies. The actual functions of differentially methylated sites in introns are not clarified yet, or whether an individual gene can be influenced by different sites within one of its introns that can be many kilobases in length. However, it is compelling that the functional categories of the genes, shown in the last column of Table S9, can be linked to the dysfunctional physiology in ME/CFS and LC, for example, transcription factors, signal transduction, calcium channels, and mitochondria. Four of the six were similarly hyper- or hypomethylated in both studies, whereas in two cases the DMF affected the methylation in opposite directions. The reason for the opposite direction could be explained by different DMFs in the intron being associated with the gene regulation. The five gene-associated genomic sites with the highest differential methylation were introns in both studies. Additionally, one gene was linked to a fragment in an exon in our study, but to an intergenic site in the Helliwell et al. (2020) study [23].

Functional pathway analysis (Figure S1A,B) using Metascape [32] revealed key shared pathways between LC and ME/CFS, with significant enrichment in immune regulation, response to wounding, cytokine signalling, and small GTPase-mediated signal transduction. These pathways align with previous findings of immune dysregulation and inflammatory responses in both conditions. However, pathway enrichment also suggested condition-specific differences that may also relate to the triggering source for the condition (SARS-CoV-2 virus for LC, and other viral triggers for ME/CFS) or the stage of the disease condition. LC showed significant associations with blood vessel morphogenesis, neutrophil degranulation, and the AGE-RAGE pathway, while ME/CFS exhibited associations with thyroid hormone production, leukocyte differentiation, and T cell receptor regulation (data shown in Table S7). These may reflect distinct differences in the immunopathological mechanisms underlying the stage of each condition, with the more juvenile LC (one year from onset in these patients) potentially involving more acute inflammatory and vascular responses, while the more mature ME/CFS (average 12 years in this age/sex matched cohort) may involve more chronic immune dysregulation mechanisms.

**Figure 5:**
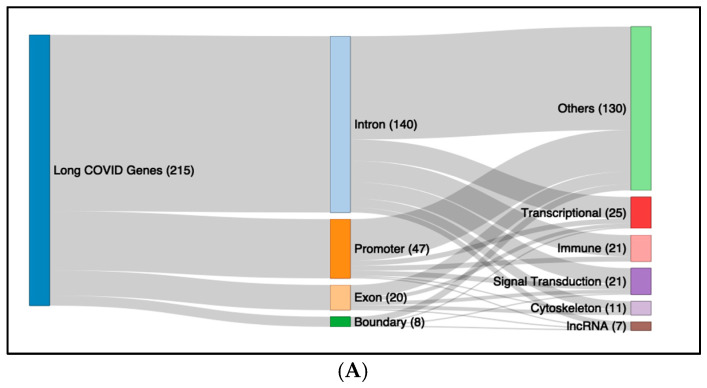

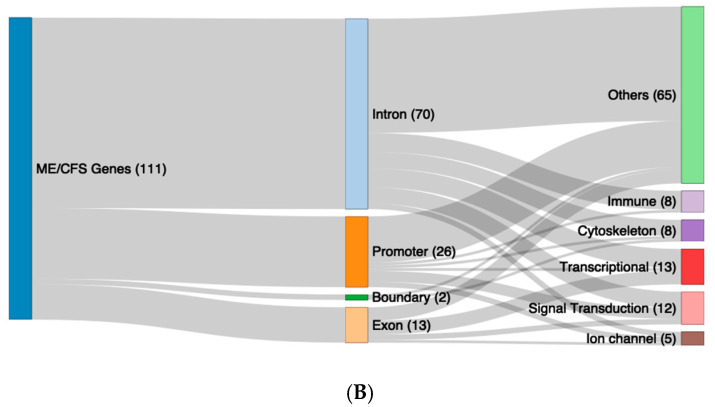
Sankey Plot showing the effects of differential methylation at regions of specific genes on molecular physiology of LC and ME/CFS. The figure demonstrates the connection of the differential methylation to gene regions of specific genes in (**A**). Long COVID and (**B**). ME/CFS. The locations of the sites within the genes (intron, exon, promoter, exon/intron boundary) and the main functional categories of molecular physiology affected, categorized by AI based on the functions derived from Gene Cards.

Using the same genes implicated in the generation of the data from Metascape, we explored their functions separately on Gene Cards and then utilised an AI tool to generate a broader functional category based on the functions derived from the Gene Cards. From this analysis, a Sankey plot was produced to give a broader overview of the consequences of the differential methylation changes exhibited in each condition, ME/CFS and LC. (Figure 5). The Sankey Plot gave four broad functional categories for both LC and ME/CFS: Transcription, Immune Function, Signal Transduction, and Cytoskeleton. LC was also associated with changes in lncRNA as a major category, whereas ME/CFS had an extra category of ion channels. These plots highlight that differential methylation for both Long COVID and ME/CFS affects similar major broad molecular physiological and biochemical pathways, affirming the similar pathophysiology of the two conditions.

Although not directly comparable to the current study, a number of studies have highlighted that methylation changes have occurred as an immediate result of the COVID infection, and these changes may be the forerunners of those present in LC. A recent review of nine such studies, albeit each with different symptom assessment criteria and with no common data analysis methodology, revealed that they still gave similar results. The authors concluded that with a more rigorous common methodology, the changes observed could be useful for the clinical management of the acute infection [53]. A study of post-acute sequelae of COVID-19 (PASC) with 103 patients spanning a 30-day period post-infection involved whole genome methylation sequencing of DNA isolated from whole blood and showed DMRs that differentiated patients from the control groups. It was concluded that this epigenetic signal also provided an opportunity to follow the PASC progression [54]. A longitudinal PASC study that could be regarded as an LC study involved 22 patients at three, six, and 12 months with DNA isolated from PBMCs, and using an Infinium Human Methylation 850 K Bead Chip. It showed a four-fold decrease in the number of DMF from three to 12 months. It was not possible to compare this with the current study here as a higher significance stringency *p* < 0.01, but no methylation differences were the limits, and the methodology was different [55]. An interesting ME/CFS/COVID crossover study used DNA methylation in ME/CFS patients and observed a downregulation of the expression of the human angiotensin-converting enzyme 2 (ACE2) used by the SARS-CoV-2 virus during infection, but not homologous ACE. High ACE:ACE2 ratios have been inferred to be an indicator of severe COVID outcomes [56].

## 4. Materials and Methods

### 4.1. The Analysis Cohorts

We analysed the DNA methylomes of five LC patients, five age/sex matched ME/CS patients, and five age/sex matched healthy controls (HC) by Reduced Representation Bisulphite Sequencing (RRBS) to identify differentially regulated DNA fragments in the three groups. The ME/CFS patients had been diagnosed using the Canadian Consensus Criteria (2003) by an expert ME/CFS clinician [57] and the LC patients from the combination of a positive test for COVID-19, and the subsequent development of symptoms indicating an ME/CFS-like syndrome [15], consistent with the WHO clinical case definition of 2021 [58]. The demographics of the three cohorts are shown in Table 3. Patients with ME/CFS and LC filled in detailed questionnaires seeking the origin and course of their illness and the symptoms associated with it. The symptoms were graded on a severity scale. All were significantly affected (moderate to serious) but were able to go to a community pathology laboratory to give their blood samples. No patient was classified as ‘severely ill’, that is, bedbound or completely housebound.

### 4.2. PBMC Isolation

Patients completed a brief survey of their health status on the day at the time of blood collection. Blood fractions were processed within 24 h. Peripheral blood mononuclear cells (PBMCs) were isolated from whole blood as described [59] by layering it onto Ficoll-Paque (Cytiva, Uppsala, Sweden), followed by centrifuging at 400× *g* to separate plasma from PBMCs and the PBMCs from other cells. The removed PBMC layer was diluted with PBS and then pelleted at 100× *g*. The pellet was washed in PBS and stored at −80 °C in a solution of FCS containing 10% DMSO.

### 4.3. DNA Extraction

DNA was extracted from 200 μL of the PBMC fraction using the Illustra Blood Genomic Prep Mini Spin Kit (GE Healthcare UK Ltd., Chalfont St Giles, Bucks, UK) following the manufacturer’s instructions. Elution was performed using the kit’s EB buffer, and DNA concentration was measured with a Qubit 2.0 fluorometer according to the Qubit dsDNA HS Assay Kit protocol ThermoFisher Scientific, Waltham, MA, USA).

### 4.4. Reduced Representation Bisulphite Sequencing

Reduced Representation Bisulphite Sequencing (RRBS) libraries were prepared following previously established protocols [60,61]. In summary, 500 µg of genomic DNA was digested using 160 U of the MspI restriction enzyme. After end repair and adenylation of the 3′ ends, adaptors were ligated to the DNA fragments. Bisulphite conversion was carried out using the EZ DNA Methylation Kit (Zymo Research Corp, Irvine, CA, USA) according to the manufacturer’s instructions. A semi-quantitative PCR was performed on the bisulphite-converted DNA to determine the optimal number of amplification cycles required for the final large-scale PCR of the complete library. Following PCR amplification, DNA was size-selected using magnetic beads (AMPure XP beads from the TruSeq DNA nanokit -San Diego Illumina, Inc., San Diego, CA, USA) as described in [58], isolating fragments between 40–220 bp to construct the RRBS libraries while minimizing adaptor contamination. The purified DNA was assessed for quality using a BioAnalyzer (Agilent, Santa Clara, CA, USA) and Qubit (ThermoFisher Scientific, Waltham, MA, USA) measurements, followed by further purification with AMPure XP Beads (Beckman Coulter, Brea, CA, USA).

#### 4.4.1. DNA Sequencing

Samples were sequenced at the Otago Genomics and Bioinformatics Facility. After sequencing, raw FASTQ files were assessed for adaptor sequences and trimmed accordingly. The processed reads were then aligned to the human genome (GRCh37/hg19) using Bismark, generating BAM files for subsequent differential methylation analysis.

#### 4.4.2. Statistical Analyses

Analyses were conducted using the updated version of the DMAP pipeline [62,63], namely the DMAP2 analysis pipeline [31] on a macOS computer, to examine methylation changes across fragments ranging from 40 to 220 bp. DMAP2 employed an ANOVA F-test to compare patient and control groups, ensuring that only fragments with data available for all individuals in each group were included. A raw *p*-value threshold of <0.05 was applied. We have used a stringent *p*-value threshold without false discovery rate correction in order not to lose true positives from this analysis, considering our sample size is low. Genomic features overlapping with these fragments were identified using the in-built Geneloc function of DMAP2. For the function of the genes, Gene Cards were used, and to assign the functional categories, ChatGPT was used to assign the functions from Gene Cards.

## 5. Conclusions

A comparative study of the DNA methylation changes in small cohorts of age/sex matched ME/CFS patients and LC patients was compared against similarly matched HCs. The RRBS sequencing data contained 342,055 fragments that covered > 2 million CpG sites across the 15 samples that were suitable for comprehensive genome-wide analysis. With the small sizes of the three cohorts, the 73,239 fragments that were present in all 15 participants were selected for further analysis. Of these, 3363 contained differentially methylated CpGs when a significance limit of *p* < 0.05 was set, but with no limit on the methylation difference. A PCA plot of the fifteen participants’ data showed that the patients of three cohorts formed tightly clustered groups in each case, which were well separated. This implied all members of each cohort, including all the HCs, had similar methylation levels of the fragments to the other members of their cohorts. The separation of the ME/CFS and LC cohorts implied either that there was a difference in the degree of change in the methylation between the ME/CFS patients and LC patients at the same sites, or that there may be methylation changes at specific positions in one of the cohorts but not the other.

When the limit of methylation change was set at >10%, 429 DMFs were found in the LC cohort and 214 DMFs in the ME/CFS cohort, with 118 fragments meeting the set limits common to both. The DMF sites were mainly hypermethylated, in a ratio of 2:1 with hypomethylation in both cohorts compared with the HCs. There was a high correlation (Pearson R score 0.88) of the common fragments in the two cohorts, emphasising the similarity between the two conditions. Unsupervised hierarchical clustering of DMFs was displayed on heat maps of ME/CFS vs. HCs and LC vs. HCs. There were DMFs in gene promoters, exons, and introns, and in the intergenic regions. Twelve of the DMFs were associated with gene promoters associated with specific genes in both ME/CFS and LC, with the changes in the same direction, except for one promoter site, where the methylation change was in opposite directions between ME/CFS and LC. Six of the DMFs were associated with gene exons and changed in the same direction in the two cohorts, except for one site where the direction of change was in the opposite direction. Box plots confirmed that in all patients of each cohort, there was a consistent shift in methylation at the DFM sites compared with HCs, and the variations among the patient shifts were generally small, including in the base levels of methylation among the HC participants. This could account for the tight clustering of the patients in each cohort observed in the PCA plot. Of the 118 DMFs in common between ME/CFS and LC, 26 had > 10% methylation change between the two cohorts, particularly when it involved hypomethylation (15 in intergenic regions and 11 in defined gene regions). In most cases, LC had the greater methylation change, and now with the inclusion of the intergenic sites, six of the 26 DMFs showed opposite methylation changes (mainly hypermethylation in LC and hypomethylation in ME/CFS).

There were 215 unique genes that could be linked to the LC DMFs and 111 genes to the ME/CFS DMFs, and pathway enrichment analysis with Metascape linked them to shared pathways in the two conditions, suggesting common biological mechanisms. Examples are tissue repair and inflammatory processes, immune signalling and inflammation, and physiological processes like circulation and metabolism. A Sankey plot was produced utilizing an AI tool to give a broader view of the consequences of the differential methylation changes in ME/CFS and LC, respectively. Of the five major functional categories identified, four were recognised for both ME/CFS and LC: transcription, immune function, signal transduction, and cytoskeleton. LC was also associated with changes in lncRNA as a major category, whereas ME/CFS had as an extra category, ion channels.

## 6. Future Directions

Since LC is a relatively recent post-viral chronic condition arising from the pandemic with a maximum time since infection of about ~5 years (in our study all 1 year) it contrasts with ME/CFS in that diagnosis of ME/CFS has generally taken several years in the absence of a molecular diagnostic test, and most patients volunteering for studies have had their condition for 10 year of more. So, the ME/CFS -like syndrome, making up ~50% of the LC patients, could be regarded as a juvenile form of ME/CFS in this study. It will be important to investigate whether the minor differences we observed in LC compared with ME/CFS relate to the stage of the illness or are derived from the specific effects of the SARS-CoV-2 virus. Importantly, now that regular testing for COVID has fallen away dramatically, and the virus has seemingly become endemic, diagnosis will not be aided by the previous positive testing. New cases of LC will be more like ME/CFS, requiring validation. This emphasises the need for an early diagnostic test that could identify both illnesses together, but also LC specifically, for best practice management of each condition. One in 20 of every new case of COVID infections (~5%) that are still occurring daily has the risk of developing LC. What is so encouraging from our study is the identification of specific changes in DNA methylation that occurred in all patients of the cohorts and were not reflected in variations of levels of methylation in the healthy controls. This suggests that molecular signatures common to both ME/CFS and LC, with high specificity and selectivity for a molecular diagnostic test, will be possible. The difference we observed between ME/CFS and LC also suggests that a specific test for LC might be possible if the differences are not simply disease-stage specific. Validation studies with larger cohorts using the same limits, methodology, and analysis tools are now required. Very few longitudinal studies have been carried out to date to follow the course of these ongoing long-term illnesses. DNA methylation analysis can provide a tool for this and may be able to provide information for better management and improve the current chaotic status of treatments for individual patients.

Tools to inform this will enhance the possibility of affected patients having a better quality of life. But how can they be made accessible to all patients and their clinicians? The need is for a test that can be ordered by clinicians for their patients and conducted in community pathology laboratories. There is hope with the development of Nanopore sequencing technology that a workflow can be developed. A well-validated DNA methylome panel will be a large, user-friendly, cost-effective sequencing assay for widespread community use for early diagnosis and in LC and ME/CFS. Such an assay is amenable to being converted into a USB-sized, portable Nanopore MinION assay that could fulfil the need.

## Figures and Tables

**Table 1:** Twenty-six DMFs segregating HC, LC, and ME/CFS cohorts from each other.

Chr	Start	End	Location	GeneID	%Difference LC v. HC	%Difference ME v. HC	%Difference LC v. ME
**18**	34854648	34854693	intron	*CELF4*	−13.9	+15.1	−29.0
1	90074598	90074699	Intergenic		−36.4	−15.7	−20.7
16	4527540	4527641	Promoter	*NMRAL1*	−32.0	−12.5	−19.5
5	39185995	39186145	intron	*FYB*	−33.3	−14.5	−18.8
2	240241154	240241261	Intergenic		+13.4	+32.2	−18.8
7	158766236	158766379	Intergenic		+18.4	+35.4	−17.0
8	61778005	61778136	exon	*CHD7*	−29.2	−14.5	−14.7
17	76129099	76129221	intron	*TMC8*	−24.7	−13.3	−11.4
6	30854000	30854160	intron	*DDR1*	−22.4	−11.0	−11.4
17	75429645	75429795	Intergenic		−21.9	−10.6	−11.3
1	59090260	59090360	Intergenic		+12.4	+22.8	−10.6
2	121955379	121955533	Intergenic		+22.3	+12.2	+10.1
16	3137553	3137716	Intergenic	*ZNF205*	+26.1	+14.7	+11.4
19	1047184	1047347	exon	*ABCA7*	+24.6	+11.8	+12.8
7	158250978	158251159	Intergenic		+28.1	+15.3	+12.8
14	55587537	55587752	Promoter	*LGALS3*	+28.5	+14.8	+13.7
15	75336231	75336352	intron	*PPCDC*	+26.5	+10.8	+15.7
8	58055165	58055309	Intergenic		+27.6	+11.7	+15.9
21	46714776	46714890	Intergenic		+31.5	+13.8	+17.7
8	58055310	58055463	Intergenic		+34.2	+15.7	+18.5
1	19110747	19110909	Intergenic		−16.7	−35.4	+18.7
17	76661321	76661487	Intergenic		+11.8	−11.3	+23.1
10	118025165	118025303	Intergenic		+13.1	−13.1	+26.2
6	36969405	36969621	Promoter	*FGD2*	+21.7	−11.1	+32.8
20	3732943	3733092	exon	*HSPA12B*	+18.3	−15.6	+33.9
3	126945870	126946029	Intergenic		+11.4	−25.9	+37.3

Chr is the chromosome number where the DMF is found, the start and end sites of the DMF in the genome are shown, the location of genomic region indicated, GENEID -deduced associated gene, % methylation differences between LC v. HC (first column), ME/CFS v. HC (second column), LC v. ME/CFS (third column). − hypomethylation, + hypermethylation % difference.

**Table 2:** Differentially methylated fragments at promoters and exons common to both ME/CFS and LC patients.

A. Promoters						
Chromosome	Start	End	Associated Genes	DM * (%) (LC vs. HC)	DM * (%) (HC vs. ME)	DM * (%) (LC vs. ME)
19	13841885	13841989	*CCDC130*	+14.2	+11.8	+2.4
14	77495636	77495807	*IRF2BPL*	−12.2	−10.2	−2.0
17	33776642	33776791	*SLFN13*	+18.1	+11.7	+6.4
16	84076941	84077080	*SLC38A8*	+12.7	+13.0	−0.3
19	36249868	36250044	*HSPB6*	+11.1	+10.3	+0.8
16	4527540	4527641	*NMRAL1*	−32.0	−12.5	−19.5
14	55587537	55587752	*LGALS3*	+28.5	+14.8	+13.7
20	57581333	57581441	*CTSZ*	+21.2	+18.2	+2.8
20	35170171	35170286	*MYL9*	+13.2	+11.6	+1.6
12	2027243	2027352	*CACNA2D4*	+17.7	+17.6	+0.1
6	36969405	36969621	*FGD2*	+21.7	−11.1	+32.8
6	31939186	31939321	*DOM3Z*	+21.4	+15.6	+5.8
B. Exons						
1	245851466	245851609	*KIF26B*	−19.0	−18.7	−0.3
8	61778005	61778136	*CHD7*	−29.2	−14.5	−14.7
1	226821736	226821914	*ITPKB*	−12.1	−13.1	+1.0
17	40463432	40463555	*STAT5A*	+16.2	+11.4	+4.8
20	3732943	3733092	*HSPA12B*	+18.3	−15.6	+33.9
19	1047184	1047347	*ABCA7*	+24.6	+11.8	+12.8

‘Start and end’ refer to the genomic numbering of the start and end of the DFM. * DM (%) is the percentage differential methylation.

**Table 3:** Age/sex of the matched ME/CFS, LC, and HC cohorts.

Patient	Age	Sex	Patient	Age	Sex	Control	Age	Sex
ME030	40	F	LC01	43	F	HC18	46	F
ME028	19	F	LC02	27	F	HC39	26	F
ME027	65	F	LC03	65	F	HC10	59	F
ME029	40	M	LC04	42	M	HC37	40	M
ME007	27	F	LC05	36	F	HC38	31	F

## Data Availability

All datasets generated and analysed during this current study are available in the GEO database NCBI -GSE297189.

## Supplementary Materials

The following supporting information can be downloaded at https://www.mdpi.com/article/10.3390/ijms26146631/s1.

## References

[1] WHO (2025). COVID-19 Cases, World. https://data.who.int/dashboards/covid19/cases.

[2] Tate W.P., Walker M.O.M., Peppercorn K., Blair A.L.H., Edgar C.D. (2023). Towards a Better Understanding of the Complexities of Myalgic Encephalomyelitis/Chronic Fatigue Syndrome and Long COVID. Int. J. Mol. Sci..

[3] Davis H.E., McCorkell L., Vogel J.M., Topol E.J. (2023). Long COVID: Major findings, mechanisms and recommendations. Nat. Rev. Microbiol..

[4] Goldowitz I., Worku T., Brown L., Fineberg H.V., National Academies of Sciences, Engineering, and Medicine, Health and Medicine Division, Board on Global Health, Board on Health Sciences Policy, Committee on Examining the Working Definition for Long COVID (2024). A Long COVID Definition: A Chronic, Systemic Disease State with Profound Consequences.

[5] Collins F.S. (2021). NIH Launches New Initiative to Study “Long COVID”. https://www.nih.gov/about-nih/who-we-are/nih-director/statements/nih-launches-new-initiative-study-long-covid.

[6] Staff M. (1957). An Outbreak of encephalomyelitis in the Royal Free Hospital Group, London, in 1955. Br. Med. J..

[7] Levine P.H., Jacobson S., Pocinki A.G., Cheney P., Peterson D., Connelly R.R., Weil R., Robinson S.M., Ablashi D.V., Salahuddin S.Z. (1992). Clinical, epidemiologic, and virologic studies in four clusters of the chronic fatigue syndrome. Arch. Intern. Med..

[8] Shepherd C., Chaudhuri A., Association M., Association M.E., Society A.N.Z.M. (2013). ME/CFS/PVFS: An Exploration of the Key Clinical Issues.

[9] ME-Pedia (2023). Millions Missing Protests. https://me-pedia.org/wiki/Millions_Missing_protests.

[10] Tate W., Walker M., Sweetman E., Helliwell A., Peppercorn K., Edgar C., Blair A., Chatterjee A. (2022). Molecular Mechanisms of Neuroinflammation in ME/CFS and Long COVID to Sustain Disease and Promote Relapses. Front. Neurol..

[11] Fang Z., Ahrnsbrak R., Rekito A. (2024). Evidence Mounts That About 7% of US Adults Have Had Long COVID. JAMA.

[12] Xie Y., Choi T., Al-Aly Z. (2024). Postacute Sequelae of SARS-CoV-2 Infection in the Pre-Delta, Delta, and Omicron Eras. N. Engl. J. Med..

[13] Das S., Taylor K., Kozubek J., Sardell J., Gardner S. (2022). Genetic risk factors for ME/CFS identified using combinatorial analysis. J. Transl. Med..

[14] Taylor K., Pearson M., Das S., Sardell J., Chocian K., Gardner S. (2023). Genetic risk factors for severe and fatigue dominant long COVID and commonalities with ME/CFS identified by combinatorial analysis. J. Transl. Med..

[15] Gentilotti E., Górska A., Tami A., Gusinow R., Mirandola M., Rodríguez Baño J., Palacios Baena Z.R., Rossi E., Hasenauer J., Lopes-Rafegas I. (2023). Clinical phenotypes and quality of life to define post-COVID-19 syndrome: A cluster analysis of the multinational, prospective ORCHESTRA cohort. eClinicalMedicine.

[16] Jason L.A., Natelson B.H., Bonilla H., Sherif Z.A., Vernon S.D., Verduzco Gutierrez M., O’Brien L., Taylor E. (2023). What Long COVID investigators can learn from four decades of ME/CFS research. Brain Behav. Immun. Integr..

[17] Horwitz L.I., Thaweethai T., Brosnahan S.B., Cicek M.S., Fitzgerald M.L., Goldman J.D., Hess R., Hodder S.L., Jacoby V.L., Jordan M.R. (2023). Researching COVID to Enhance Recovery (RECOVER) adult study protocol: Rationale, objectives, and design. PLoS ONE.

[18] Komaroff A.L., Lipkin W.I. (2021). Insights from myalgic encephalomyelitis/chronic fatigue syndrome may help unravel the pathogenesis of postacute COVID-19 syndrome. Trends Mol. Med..

[19] Marshall-Gradisnik S., Eaton-Fitch N. (2022). Understanding myalgic encephalomyelitis. Science.

[20] Komaroff A.L. (2022). MECFS and Long COVID: Emerging Similarities and Why It Matters. https://www.youtube.com/watch?v=AbVMvRS-a7Yc.

[21] Vernon S.D., Hartle M., Sullivan K., Bell J., Abbaszadeh S., Unutmaz D., Bateman L. (2023). Post-exertional malaise among people with long COVID compared to myalgic encephalomyelitis/chronic fatigue syndrome (ME/CFS). Work.

[22] Peppercorn K., Edgar C.D., Kleffmann T., Tate W.P. (2023). A pilot study on the immune cell proteome of long COVID patients shows changes to physiological pathways similar to those in myalgic encephalomyelitis/chronic fatigue syndrome. Sci. Rep..

[23] Eaton-Fitch N., Rudd P., Er T., Hool L., Herrero L., Marshall-Gradisnik S. (2024). Immune exhaustion in ME/CFS and long COVID. JCI Insight.

[24] Helliwell A.M., Sweetman E.C., Stockwell P.A., Edgar C.D., Chatterjee A., Tate W.P. (2020). Changes in DNA methylation profiles of myalgic encephalomyelitis/chronic fatigue syndrome patients reflect systemic dysfunctions. Clin. Epigenetics.

[25] Helliwell A.M., Stockwell P.A., Edgar C.D., Chatterjee A., Tate W.P. (2022). Dynamic Epigenetic Changes during a Relapse and Recovery Cycle in Myalgic Encephalomyelitis/Chronic Fatigue Syndrome. Int. J. Mol. Sci..

[26] Brenu E.W.S., Staines D.R., Marshall-Gradisnik S.M. (2014). Methylation Profile of CD4+ T Cells in Chronic Fatigue Syndrome/Myalgic Encephalomyelitis. J. Clin. Cell. Immunol..

[27] de Vega W.C., Vernon S.D., McGowan P.O. (2014). DNA methylation modifications associated with chronic fatigue syndrome. PLoS ONE.

[28] de Vega W.C., McGowan P.O. (2017). The epigenetic landscape of myalgic encephalomyelitis/chronic fatigue syndrome: Deciphering complex phenotypes. Epigenomics.

[29] de Vega W.C., Erdman L., Vernon S.D., Goldenberg A., McGowan P.O. (2018). Integration of DNA methylation & health scores identifies subtypes in myalgic encephalomyelitis/chronic fatigue syndrome. Epigenomics.

[30] Herrera S., de Vega W.C., Ashbrook D., Vernon S.D., McGowan P.O. (2018). Genome-epigenome interactions associated with Myalgic Encephalomyelitis/Chronic Fatigue Syndrome. Epigenetics.

[31] Stockwell P.A., Rodger E.J., Gimenez G., Morison I.M., Chatterjee A. (2024). DMAP2: A Pipeline for Analysis of Whole-Genome-Scale DNA Methylation Sequencing Data. Curr. Protoc..

[32] Zhou Y., Zhou B., Pache L., Chang M., Khodabakhshi A.H., Tanaseichuk O., Benner C., Chanda S.K. (2019). Metascape provides a biologist-oriented resource for the analysis of systems-level datasets. Nat. Commun..

[33] Gopalan S., Carja O., Fagny M., Patin E., Myrick J.W., McEwen L.M., Mah S.M., Kobor M.S., Froment A., Feldman M.W. (2017). Trends in DNA Methylation with Age Replicate Across Diverse Human Populations. Genetics.

[34] Marcogliese P.C., Shashi V., Spillmann R.C., Stong N., Rosenfeld J.A., Koenig M.K., Martínez-Agosto J.A., Herzog M., Chen A.H., Dickson P.I. (2018). IRF2BPL Is Associated with Neurological Phenotypes. Am. J. Hum. Genet..

[35] Zang W., Zheng X. (2020). Structure and functions of cellular redox sensor HSCARG/NMRAL1, a linkage among redox status, innate immunity, DNA damage response, and cancer. Free Radic. Biol. Med..

[36] Di Gregoli K., Somerville M., Bianco R., Thomas A.C., Frankow A., Newby A.C., George S.J., Jackson C.L., Johnson J.L. (2020). Galectin-3 Identifies a Subset of Macrophages With a Potential Beneficial Role in Atherosclerosis. Arterioscler. Thromb. Vasc. Biol..

[37] Kuht H.J., Han J., Maconachie G.D.E., Park S.E., Lee S.T., McLean R., Sheth V., Hisaund M., Dawar B., Sylvius N. (2020). SLC38A8 mutations result in arrested retinal development with loss of cone photoreceptor specialization. Hum. Mol. Genet..

[38] Yang J.Y., Deng X.Y., Li Y.S., Ma X.C., Feng J.X., Yu B., Chen Y., Luo Y.L., Wang X., Chen M.L. (2018). Structure of Schlafen13 reveals a new class of tRNA/rRNA- targeting RNase engaged in translational control. Nat. Commun..

[39] Kanno T., Lin W.D., Fu J.L., Matzke A.J.M., Matzke M. (2017). A genetic screen implicates a CWC16/Yju2/CCDC130 protein and SMU1 in alternative splicing in Arabidopsis thaliana. Rna.

[40] Dreiza C.M., Komalavilas P., Furnish E.J., Flynn C.R., Sheller M.R., Smoke C.C., Lopes L.B., Brophy C.M. (2010). The small heat shock protein, HSPB6, in muscle function and disease. Cell Stress Chaperones.

[41] Krueger S., Bernhardt A., Kalinski T., Baldensperger M., Zeh M., Teller A., Adolf D., Reinheckel T., Roessner A., Kuester D. (2013). Induction of premalignant host responses by cathepsin x/z-deficiency in Helicobacter pylori-infected mice. PLoS ONE.

[42] Kumar C.C., Mohan S.R., Zavodny P.J., Narula S.K., Leibowitz P.J. (1989). Characterization and differential expression of human vascular smooth muscle myosin light chain 2 isoform in nonmuscle cells. Biochemistry.

[43] Qin N., Yagel S., Momplaisir M.L., Codd E.E., D’Andrea M.R. (2002). Molecular cloning and characterization of the human voltage-gated calcium channel alpha(2)delta-4 subunit. Mol. Pharmacol..

[44] Huber C., Mårtensson A., Bokoch G.M., Nemazee D., Gavin A.L. (2008). FGD2, a CDC42-specific exchange factor expressed by antigen-presenting cells, localizes to early endosomes and active membrane ruffles. J. Biol. Chem..

[45] Shen Y., Zhang J., Calarco J.A., Zhang Y. (2014). EOL-1, the homolog of the mammalian Dom3Z, regulates olfactory learning in C. elegans. J. Neurosci..

[46] Apicco D.J., Shlevkov E., Nezich C.L., Tran D.T., Guilmette E., Nicholatos J.W., Bantle C.M., Chen Y., Glajch K.E., Abraham N.A. (2021). The Parkinson’s disease-associated gene ITPKB protects against α-synuclein aggregation by regulating ER-to-mitochondria calcium release. Proc. Natl. Acad. Sci. USA.

[47] Uchiyama Y., Sakaguchi M., Terabayashi T., Inenaga T., Inoue S., Kobayashi C., Oshima N., Kiyonari H., Nakagata N., Sato Y. (2010). Kif26b, a kinesin family gene, regulates adhesion of the embryonic kidney mesenchyme. Proc. Natl. Acad. Sci. USA.

[48] Reddy N.C., Majidi S.P., Kong L., Nemera M., Ferguson C.J., Moore M., Goncalves T.M., Liu H.K., Fitzpatrick J.A.J., Zhao G. (2021). CHARGE syndrome protein CHD7 regulates epigenomic activation of enhancers in granule cell precursors and gyrification of the cerebellum. Nat. Commun..

[49] Lin J.X., Leonard W.J. (2000). The role of Stat5a and Stat5b in signaling by IL-2 family cytokines. Oncogene.

[50] Kim W.S., Weickert C.S., Garner B. (2008). Role of ATP-binding cassette transporters in brain lipid transport and neurological disease. J. Neurochem..

[51] Dib S., Pahnke J., Gosselet F. (2021). Role of ABCA7 in Human Health and in Alzheimer’s Disease. Int. J. Mol. Sci..

[52] Steagall R.J., Rusiñol A.E., Truong Q.A., Han Z. (2006). HSPA12B is predominantly expressed in endothelial cells and required for angiogenesis. Arterioscler. Thromb. Vasc. Biol..

[53] Przybylowicz P.K., Klepinowski T., Taryma-Leśniak O., Bińkowski J., Wojdacz T.K. (2025). Evaluation of methylation changes in blood cells of COVID-19 patients as a biomarker of severity of the infection. BMC Infect. Dis..

[54] Balnis J., Madrid A., Drake L.A., Vancavage R., Tiwari A., Patel V.J., Ramos R.B., Schwarz J.J., Yucel R., Singer H.A. (2024). Blood DNA methylation in post-acute sequelae of COVID-19 (PASC): A prospective cohort study. EBioMedicine.

[55] Granvik C., Persson I.-L., Barros G.W.F., Ahlm C., Forsell M.N.E., Tevell S., Sundh J., Blomberg A., Lind A., Cajander S. (2025). Long-Term Physical Capacity Following COVID-19: A Prospective, Three-Year Study. medRxiv.

[56] Pagliaro P., Penna C. (2020). ACE/ACE2 Ratio: A Key Also in 2019 Coronavirus Disease (Covid-19)?. Front. Med..

[57] Carruthers B.M., Jain A.K., De Meirleir K.L., Peterson D.L., Klimas N.G., Lerner A.M., Bested A.C., Flor-Henry P., Joshi P., Powles A.C.P. (2003). Myalgic Encephalomyelitis/Chronic Fatigue Syndrome. J. Chronic Fatigue Syndr..

[58] WHO (2021). A Clinical Case Definition of Post COVID-19 Condition by a Delphi Consensus. https://www.who.int/publications/i/item/WHO-2019-nCoV-Post_COVID-19_condition-Clinical_case_definition-2021.1.

[59] Peppercorn K., Edgar C., Al Momani S., Rodger E.J., Tate W.P., Chatterjee A. (2025). Application of DNA Methylome Analysis to Patients with ME/CFS. Methods Mol. Biol..

[60] Ludgate J.L., Wright J., Stockwell P.A., Morison I.M., Eccles M.R., Chatterjee A. (2017). A streamlined method for analysing genome-wide DNA methylation patterns from low amounts of FFPE DNA. BMC Med. Genomics.

[61] Rodger E.J., Stockwell P.A., Almomani S., Eccles M.R., Chatterjee A. (2023). Protocol for generating high-quality genome-scale DNA methylation sequencing data from human cancer biospecimens. STAR Protoc..

[62] Stockwell P.A., Chatterjee A., Rodger E.J., Morison I.M. (2014). DMAP: Differential methylation analysis package for RRBS and WGBS data. Bioinformatics.

[63] Chatterjee A., Stockwell P.A., Rodger E.J., Morison I.M. (2012). Comparison of alignment software for genome-wide bisulphite sequence data. Nucleic. Acids. Res..

