# Supplementary material for: Comparing DNA Methylation Landscapes in Peripheral Blood from Myalgic Encephalomyelitis/Chronic Fatigue Syndrome and Long COVID Patients
Source: Int J Mol Sci. 2025 Jul 10;26(14):6631. doi: 10.3390/ijms26146631 (PMC12294161; doi:10.3390/ijms26146631)
Supplement: Supplementary file 1 [file ijms-26-06631-s001.zip › Figure S1.pdf]

A. Genes related to Long COVID DMFs

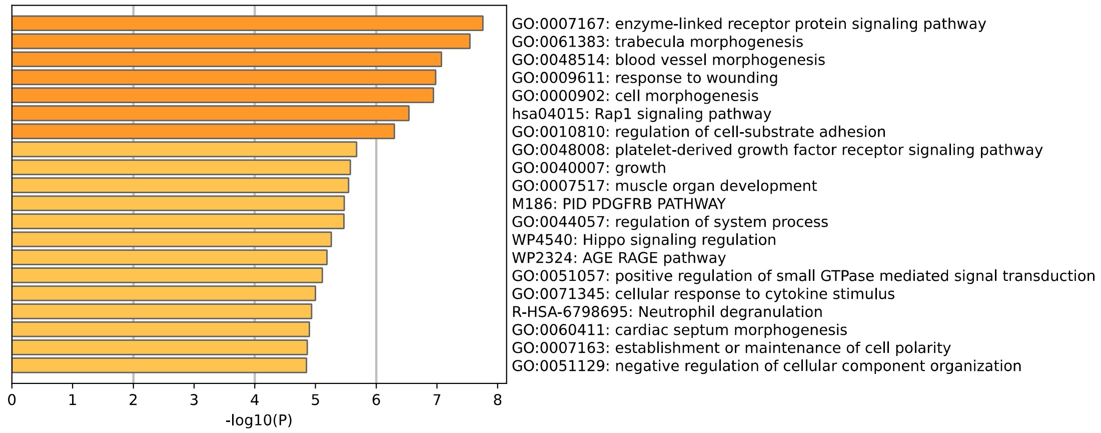

B. Genes related to ME/CFS DMFs

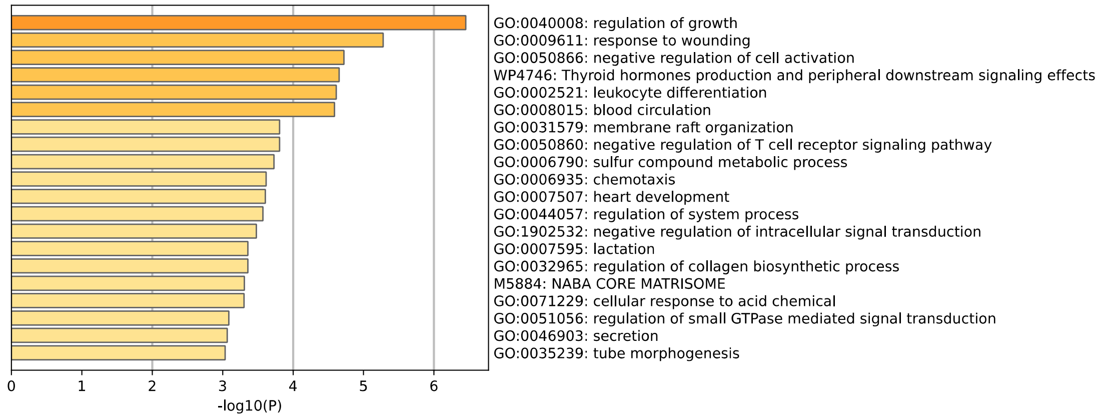

Figure S1A & S1B: Metascape Gene Enrichment Analysis for LC and ME/CFS genes associated with the DMFs excluding Intergenic sites

The figure shows the functional categories of the genes of **A**. LC and **B**. ME/CFS using Metascape [31]. The top 20 pathways are arranged based on the -log10(P) value using the Metascape server. In the case of LC, for the top 20 functional pathways, a -log10(P) value > 4 was observed whereas in the case of ME/CFS, the log10(P) value was > 3.
